# Epidemiology and serotype distribution of *Streptococcus pneumoniae* carriage among influenza-like illness cases in metropolitan Vientiane, Lao PDR: a community-based cohort study

**DOI:** 10.3389/fpubh.2023.1124016

**Published:** 2023-04-20

**Authors:** Valentina Sanchez Picot, Inthalaphone Keovichith, Phimpha Paboriboune, Bruno Flaissier, Mitra Saadatian-Elahi, James W. Rudge

**Affiliations:** ^1^Fondation Mérieux, Lyon, France; ^2^Center of Infectiology Christophe Mérieux of Laos, Vientiane, Laos; ^3^Fondation Mérieux, Vientiane, Laos; ^4^Service Hygiène, Epidémiologie et Prévention, Centre Hospitalier Hôpital Eduard Herriot, Hospices Civils de Lyon, Lyon, France; ^5^CIRI, Centre International de Recherche en Infectiologie, Team Public Health, Epidemiology and Evolutionary Ecology of Infectious Diseases (PHE3ID), University Lyon, Inserm, U1111, Université Claude Bernard Lyon 1, CNRS, UMR5308, ENS de Lyon, Lyon, France; ^6^Communicable Diseases Policy Research Group, Department of Global Health and Development, London School of Hygiene and Tropical Medicine, London, United Kingdom; ^7^Faculty of Public Health, Mahidol University, Bangkok, Thailand

**Keywords:** *Streptococcus pneumoniae*, Lao PDR, influenza-like illness, cohort study, surveillance, pneumococcal vaccine, serotypes

## Abstract

**Background:**

Data on the epidemiology of *Streptococcus pneumoniae* among influenza-like illness (ILI) cases, particularly in low- and middle-income countries are scarce. This study assessed the prevalence, risk factors and serotype distribution of *S. pneumoniae* carriage among ILI cases in metropolitan Vientiane, Lao People's Democratic Republic. The 13-valent pneumococcal conjugate vaccine (PCV13) was introduced among infants in October 2013.

**Methods:**

Active ILI surveillance was conducted through weekly phone calls in an open community-based cohort study (April 2015–February 2019), involving 5,690 participants from 1,142 randomly selected households. Participants reporting ILI symptoms provided a nasopharyngeal swab and answered a questionnaire. *S. pneumoniae* and serotype pneumococcal-positive samples were screened by Multiplex PCR assays. Chi-squared tests and generalized linear mixed models were used to test for variables associated with pneumococcal positivity.

**Results:**

Among 1,621 ILI episodes, 269 (16.6%) tested positive for nasopharyngeal pneumococcal carriage, with the highest prevalence (55.4%) in children under 5 years. Pneumococcal carriage was significantly associated with concurrent detection of *Hemophilus influenzae* (adjusted odds ratio [aOR]: 6.93; 95% CI: 2.10–22.9) and exposure to household cigarette smoke (aOR: 1.65; 95% CI: 1.07–2.54). PCV13 serotypes accounted for 37.8% of all pneumococcal isolates. Detection of PCV13 serotypes among ILI cases aged under 5 years declined significantly between 2015/16 and 2018/19.

**Conclusions:**

Community-based surveillance of *S. pneumoniae* among ILI cases complement surveillance at healthcare facilities to provide a more complete picture of pneumococcal carriage. Our findings contribute also to the growing body of evidence on the effects of PCV13 introduction on circulating serotypes and their potential replacement.

## 1. Introduction

*Streptococcus pneumoniae* is a major cause of community-acquired pneumonia and meningitis worldwide, particularly in low- and middle-income countries (1). Although all age groups are affected, the highest incidence rates of pneumococcal disease occur in young children and older adults (1, 2). Pneumococcal bacteria also frequently colonize the respiratory tract of asymptomactic carriers. Nasal carriage and related pneumococcal disease have been shown to be influenced by the presence of respiratory viruses, particularly during influenza seasons (3, 4).

Prevention of pneumococcal pneumonia involves the 23-valent pneumococcal polysaccharides vaccines (PPSV23) for adults and pneumococcal conjugate vaccines (PCV) for children. Lao PDR introduced the 13-valent PCV (PCV13) into the national childhood vaccination program in October 2013, using a 3 + 0 schedule at 6, 10 and 14 weeks of age (5). Catch-up vaccination was offered to infants up to 12 months old during the first roll-out. National coverage of the third dose of PCV13 among infants was estimated at 89% in 2020 (6).

Most previous studies on *S. pneumoniae* burden and serotype distribution in LMICs, including in Lao PDR, have been based on healthcare facility surveillance in infants and young children (5, 7, 8). To better understand *S. pnuemoniae* epidemiology and transmission, including risk factors for carriage or infection, and the impact of vaccination on circulating serotypes, prospective community-based studies, across all age groups, are also needed.

The longitudinal surveillance study of respiratory infectious agents in metropolitan Vientiane, Lao PDR (LaCoRIS) is a community-based cohort study that monitored the etiology and burden of influenza-like illness among residents of this region from 2015 to 2019. The findings of the first year of surveillance, covering over 20 respiratory pathogens, have been published elsewhere (9), with *S. pneumoniae* the most comon bacterial agent detected in cases of influenza-like illness (ILI) (9). In this paper, we focus on *S. pneumoniae* as the main outcome of interest, to report on prevalence, risk factors and serotype distribution of pneumococcal carriage among ILI cases in the LaCoRIS study during the three surveillance years.

## 2. Methods

Full details on the LaCoRIS study design, including recruitment and surveillance of households in the cohort, are reported elsewhere (9). In brief, participants were recruited from randomly selected households in 25 villages in metropolitan Vientiane. Participant enrolment into the cohort was conducted at two main timepoints. The first enrolment phase began in April 2015, during which a total of 4,855 individuals from 995 households were recruited and followed-up for ~1 year (until 31 May 2016), as reported previously (9). The cohort study then resumed in March 2017 (i.e., approximately a year after the end of the first surveillance period), with a second enrolment phase conducted to initiate a further 2 years of ILI surveillance. For this second phase, all previously recruited households were invited to participate, along with additional, randomly selected households within the same villages, to account for loss of follow-up. Thus, ILI surveillance was conducted among an open cohort over three surveillance years i.e., year 1 (from 28/04/2015 to 31/05/2016), year 2 (from 01/03/2017 to 28/02/2018), and year 3 (from 01/03/2018 to 28/02/2019).

During these surveillance periods, active case finding for ILI was conducted through weekly phone calls to each participating household. Individuals fulfilling the WHO case definition for ILI were visited to administer a questionnaire and collect a nasopharyngeal swab, after obtaining informed written consent (National Ethics Committee 060/NECHR; 1 January 2017). Follow-up questionnaires were administered to ILI cases ~40 days after the sample collection and case investigation, to obtain information on clinical outcomes (e.g., hospitalization or sequelae) that had since occurred in relation to the ILI episode.

The FTD^®^ Respiratory pathogens 21 Plus technology was used for real-time Polymerase Chain Reaction (qPCR) testing of swabs, as detailed previously (9). Detection of *S. pneumoniae* with this assay targets the *lyt A* gene, a largely conserved gene encoding the major autolytic enzyme of pneumococcus. This approach is considered a gold standard among culture-independent *S. pneumoniae* assays, with reported sensitivity and specificity of 100 and 99.5%, respectively (10), with a detection limit of one plasmid copy per 10μl reaction (11). *S. pneumoniae*-positive samples were then screened for 40 different serotypes using a multiplex qPCR assay, following published protocols (12). This assay has shown high sensitivity for the included serotypes, able to detect < 100 colony forming units (CFU) per reaction for the majority (91%) of strains tested (12).

All statistical analyses were conducted in R version 4.2.2. Incidence of ILI and 95% confidence intervals (CI) were estimated using complex survey analysis methods, adjusting for the demographic structure (age and sex) of the Vientiane urban population and clustering in the survey design, as detailed previously (9, 13). The prevalence of pneumococcal detection among ILI cases, and the proportional distribution of serotype categories among pneumococcal-positive ILI cases, were calculated and assessed for associations with age-group, surveillance year, and climatic season (wet vs. dry months) using chi-squared tests. Binomial generalized linear mixed models (GLMMs), adjusting for random effects at household-level, were used to further investigate risk factors for pneumococcal positivity among ILI cases using the “lme4” R package (14). Bivariable GLMM analyses tested for associations with each independent variable separately, while multivariable GLMMs included age group, sex, and any covariates which showed associations with *P* < 0.05 in the bivariable models. We were unable to adjust for individual level random effects in these GLMMs as the majority of study subjects with ILI experienced only a single episode throughout the surveillance period (Models specifying two nested levels of random effects, i.e., for both households and individuals, displayed poor convergence and singularity issues, indicating that these were overly complex for the dataset). However, to assess whether the results of our GLMM analyses may have been influenced by individual-level random effects which could not be adjusted for in the models, we also repeated these analyses with a subset of the data which included only a single ILI episode per subject. Simpson's Diversity Indices with 95% Cis were calculated using the “simboot” package (15) to assess for differences in diversity of serotypes between age groups and surveillance years, following Løchen et al. (16).

## 3. Results

Throughout the study period, a total of 1,142 households (5,690 individuals) were recruited. Of 995 households (4,885 individuals) enrolled in the first surveillance period (2015/16) (9), 936 households (94.1%) enrolled for the second phase (2017–19), along with an additional 147 new households (765 individuals). The mean duration of ILI surveillance for each household was 148 weeks (range 51–141 weeks), with a total follow-up size of 16,104 person-years across all households and surveillance years.

### 3.1. Incidence of ILI

During the three surveillance years, 1,621 ILI episodes were reported among 1,116 (19.6%) individuals from 613 (53.6%) households. The mean age of ILI cases was 37 years (range 1–100), with 74 (4.6%) and 316 (19.5%) of cases aged < 5 years and 5–15 years, respectively. Over half (970; 59.8%) of the ILI cases were females. The estimated incidence of ILI, adjusted for participant follow-up time and the demographic structure of the Vientiane population, was 10.0 (95% CI: 9.1–11.0) episodes per 100 person-years.

### 3.2. Prevalence and risk factors for pneumococcal carriage

**Figure 1:**
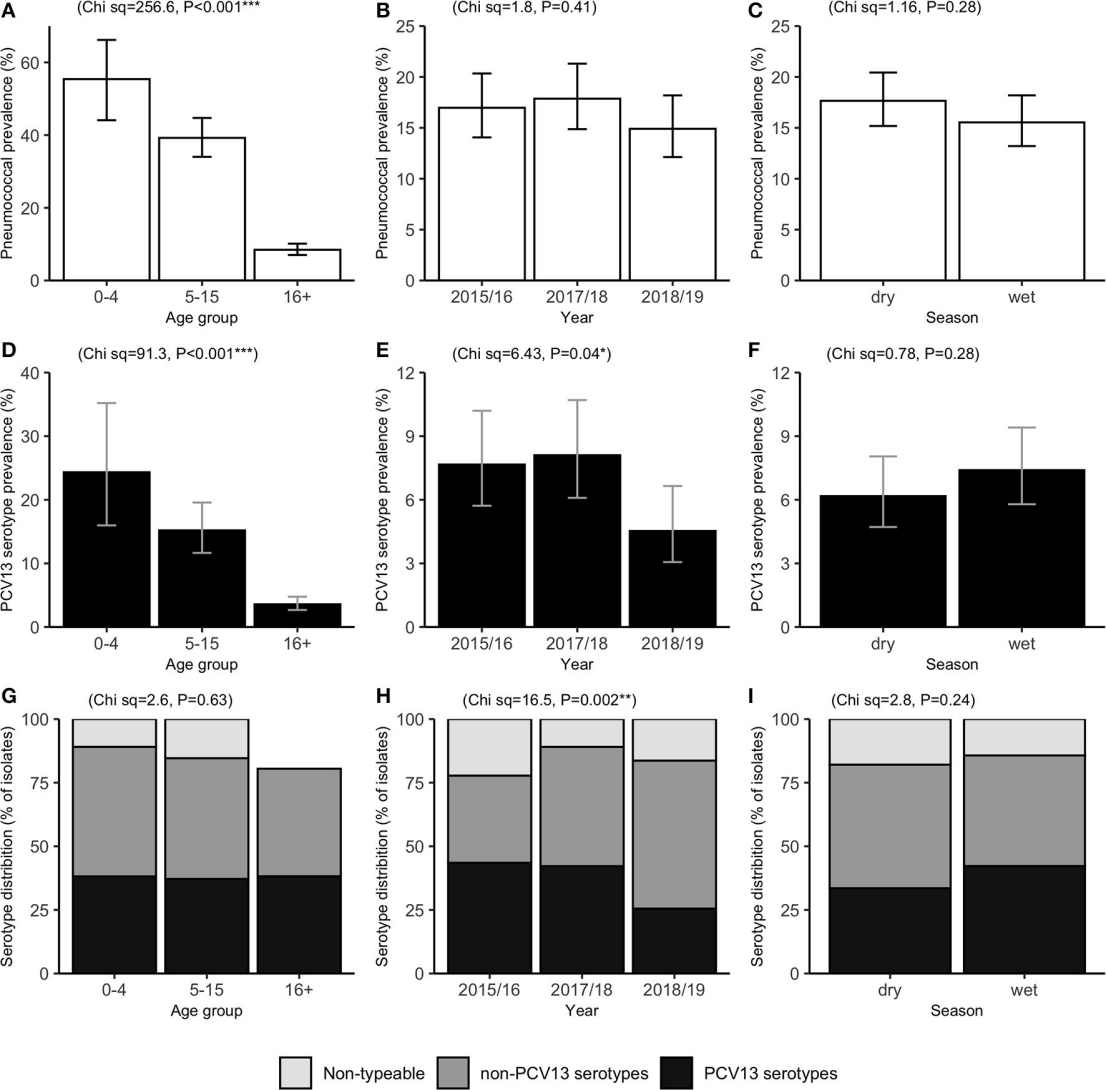
Prevalence of pneumococcal carriage **(A–C)** and PCV13 serotype carriage **(D–F)**, and the percentage of pneumococcal isolates by serotype category **(G–I)**. Results are shown among influenza-like illness cases (*n* = 1,621), by age group (left), surveillance period (middle), and season (right). PCV13: 13-valent pneumococcal conjugate vaccine. Error bars represent exact 95% confidence intervals. Chi-squared (Ch sq) statistics and *P*-values for association with x-axis levels are shown at the top of each plot.

Among 1,621 ILI episodes, 269 (16.6%) tested positive for presence of nasopharyngeal *S. pneumoniae*. The highest prevalence was observed in children under 5 years (55.4%), followed by those aged 5–15 years (39.2%), with relatively low prevalence observed in cases aged over 16 years (8.4%) (${\chi^{2}}_{2}=$ 256.6, *P* < 0.001) (Table 1, Figure 1A). In addition to strong associations with age, pneumococcal carriage was significantly associated with concurrent nasopharyngeal detection of *Hemophilus influenzae* (adjusted odds ratio [aOR]: 6.93; 95% CI: 2.10–22.9) and exposure to household cigarette smoke (aOR: 1.65; 95% CI: 1.07–2.54) in both bivariable and multivariable GLMM analyses (Table 1). No significant associations were observed with variables such as surveillance year, season, sex, socio-economic status, or respiratory virus co-infections. Household “crowding” (number of persons per sleeping room), and the number of children within the household, were significantly associated with pneumococcal carriage in bivariable analyses, but not after adjusting for other covariates. GLMM analyses conducted on a subset of the data, which excluded data from repeat ILI episodes within the same individuals, identified very similar bivariable and multivariable associations (Supplementary Table 1). This suggests that the presence of any individual-level random effects, which we were unable to adjust for in the GLMMs (see Methods), did not substantially bias or otherwise influence the associations shown in Table 1.

**Table 1:** Bivariable and multivariable analyses for associations with nasopharyngeal pneumococcal detection among influenza-like illness cases (*n* = 1,621).

			**Bivariable analysis**		**Multivariable analysis**	
**Variable**	**Level**	**Pneumococcal carriage n/N (%)**	**Crude OR**^a^ **(95% CI)**	***P***	**Adjusted OR**^a^ **(95% CI)**	***P***
Age group (y)	0-4	41/74 (55.41)	ref		ref	
	5-15	124/316 (39.24)	0.34 (0.16 to 0.70)	0.003	0.34 (0.16 to 0.74)	0.006
	16+	104/1231 (8.45)	0.03 (0.02 to 0.07)	< 0.001	0.04 (0.02 to 0.09)	< 0.001
Sex	Female	155/970 (15.98)	ref		ref	
	Male	114/651 (17.51)	1.20 (0.86 to 1.69)	0.285	1.06 (0.74 to 1.53)	0.745
Year	2015/16	93/548 (16.97)	ref			
	2017/18	97/543 (17.86)	1.08 (0.72 to 1.60)	0.713		
	2018/19	79/530 (14.91)	0.80 (0.53 to 1.21)	0.289		
Season	Dry	143/810 (17.65)	ref			
	Wet	126/811 (15.54)	0.77 (0.55 to 1.08)	0.130		
Residence area	Urban	53/359 (14.76)	ref			
	Peri-urban	90/584 (15.41)	0.89 (0.49 to 1.60)	0.692		
	Suburban	126/678 (18.58)	1.35 (0.77 to 2.36)	0.288		
SES category	Lowest	85/517 (16.44)	ref			
	Medium	74/553 (13.38)	0.81 (0.47 to 1.38)	0.439		
	Highest	110/551 (19.96)	1.53 (0.91 to 2.57)	0.107		
HH crowding (Persons per sleeping room	< 2.5	157/1120 (14.02)	ref		ref	
	≥2.5	112/501 (22.36)	2.07 (1.30 to 3.30)	0.002	1.41 (0.89 to 2.25)	0.146
No. of children < 5y in HH	0-1	247/1534 (16.10)	ref		ref	
	2 or more	22/87 (25.29)	3.00 (1.22 to 7.40)	0.017	1.01 (0.41 to 2.53)	0.978
No. of children 5-15y in HH	0-1	160/1185 (13.50)	ref		ref	
	2 or more	109/436 (25.00)	2.40 (1.50 to 3.82)	< 0.001	1.36 (0.84 to 2.22)	0.214
Exposure to HH cigarette smoke	No	116/873 (13.29)	ref		ref	
	Yes	153/748 (20.45)	1.75 (1.14 to 2.67)	0.010	1.65 (1.07 to 2.54)	0.023
Chronic condition	No	240/1363 (17.61)	ref			
	Yes	29/258 (11.24)	0.59 (0.34 to 1.03)	0.062		
*S. aureus*	No	231/1420 (16.27)	ref			
	Yes	38/201 (18.91)	1.28 (0.79 to 2.08)	0.315		
*C. pneumoniae*	No	264/1610 (16.39)	ref		ref	
	Yes	5/11 (45.45)	6.53 (1.32 to 32.2)	0.021	2.40 (0.49 to 11.8)	0.279
*M. pneumoniae*	No	261/1593 (16.38)	ref			
	Yes	8/28 (28.57)	2.49 (0.84 to 7.41)	0.101		
*H. influenzae*	No	254/1597 (15.90)	ref		ref	
	Yes	15/24 (62.50)	20.7 (6.31 to 68.2)	< 0.001	6.93 (2.10 to 22.9)	0.001
Respiratory virus (any)	No	75/492 (15.24)	ref			
	Yes	194/1129 (17.18)	1.17 (0.82 to 1.68)	0.381		
Influenza A	No	235/1397 (16.82)	ref			
	Yes	34/224 (15.18)	0.88 (0.54 to 1.43)	0.602		
Influenza B	No	242/1482 (16.33)	ref			
	Yes	27/139 (19.42)	1.14 (0.64 to 2.02)	0.665		
Coronavirus (seasonal)	No	230/1386 (16.59)	ref			
	Yes	39/235 (16.59)	1.08 (0.68 to 1.72)	0.757		
Rhinovirus	No	216/1290 (16.74)	ref			
	Yes	53/331 (16.01)	0.98 (0.66 to 1.47)	0.938		
Parainfluenza	No	244/1488 (16.39)	ref			
	Yes	25/133 (18.79)	1.44 (0.81 to 2.57)	0.216		

^a^OR, odds ratio. Crude ORs are adjusted for random effects at household level. Adjusted ORs are derived from a generalized linear mixed model which included age, sex, and all covariates which showed significance of P < 0.05 in bivariable analyses (i.e., adjusting for all the other covariates in the Table for which adjusted ORs are shown, in additional to random effects at household level).

### 3.3. Prevalence and distribution of pneumococcal serotypes

**Figure 2:**
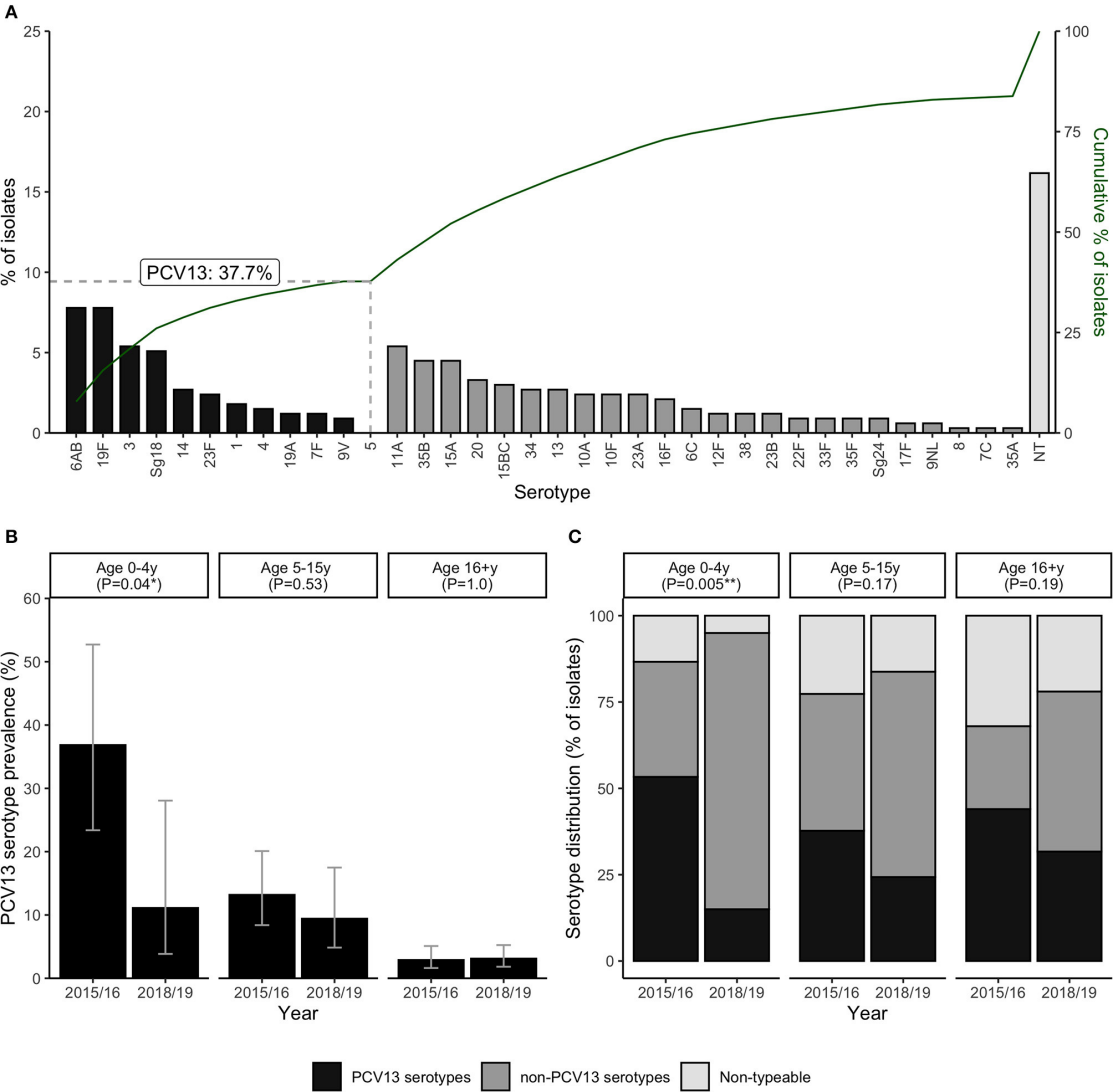
Carriage of pneumococcal vaccine (PCV13) and non-vaccine serotypes among ILI cases (*n* = 1,621). Panels show the percentage distribution and cumulative distribution of pneumococcal isolates by individual serotype **(A)**; PCV13 serotype carriage by age group and surveillance year **(B)**; and the percentage of pneumococcal isolates in each serotype category by age group and surveillance year **(C)**. *P*-values in panels **B, C** are derived from Chi-squared tests for differences between surveillance years. Error bars in panel B represent exact 95% confidence intervals.

Serotyping data was successfully obtained from 215 (79.9%) of the pneumococcal-positive cases; the remainder were classified as carrying non-typeable (NT) isolates. Carriage of at least one vaccine serotype (VT), defined as any serotype included in the PCV13 vaccine, was detected in 110 cases, corresponding to 6.8% of all ILI episodes and 40.9% of pneumococcal-positive ILI episodes. The percentage and cumulative percentage of pneumococcal isolates by each serotype are shown in Figure 2A. VT isolates accounted for 126 (37.8%) of all pneumococcal isolates (including NT isolates) detected across ILI specimens. The most commonly detected VT serotypes were 6AB, 19F, 3 and Sg18, while no isolates of serotype 5 were detected. Of the 28 non-vaccine serotypes (NVTs) included in the serotyping PCR assay, 24 were detected in at least one ILI episode, with the most common NVTs being 11A, 35B, 15A, and 20. Simpson's diversity indices did not indicate any significant differences in serotype diversity between age groups or surveillance years (data not shown).

The percentage distributions of isolates by serotype category (VT, NVT, and NT) were very similar across age groups (${\chi^{2}}_{4}=$ 2.6, *P* = 0.63) (Figure 1). However, there was evidence of a temporal change in VT carriage (${\chi^{2}}_{2}$ = 6.4, *P* = 0.04), which fell from 7.6 to 8.1% in Years 2015/16 and 2017/18, to 4.5% in 2018/19. This trend was also observed in the distribution of isolates by serotype category (${\chi^{2}}_{4}=$ 16.5, *P* = 0.002), with VT accounting for over 40% of isolates in 2015/16 and 2017/18, and only 25.5% in 2018/19 (Figure 1).

Further analyses were carried out to investigate whether this decrease in prevalence of VT detection over the study period was age-group specific (Figures 2B, C). Data from the second surveillance year (2017/18) were excluded from these age-stratified analyses, due to the low sample size of children under 5y during this period (only 9 ILI cases, of which 3 were pneumococcal-positive). Notably, the decrease in VT detection between 2015/16 and 2018/9 was much more pronounced, and only statistically significant, in children under 5y. In this age group, prevalence of VT detection fell from 36.8% (14/38) to 11.1% (3/27) (${\chi^{2}}_{1}=$ 1.2, *P* = 0.04) (Figure 2B), and the percentage of isolates identified as VT serotypes fell from 55.3% to 15.0% (${\chi^{2}}_{1}=$ 10.5, *P* = 0.005) (Figure 2C).

### 3.4. Serotype co-detection

**Figure 3:**
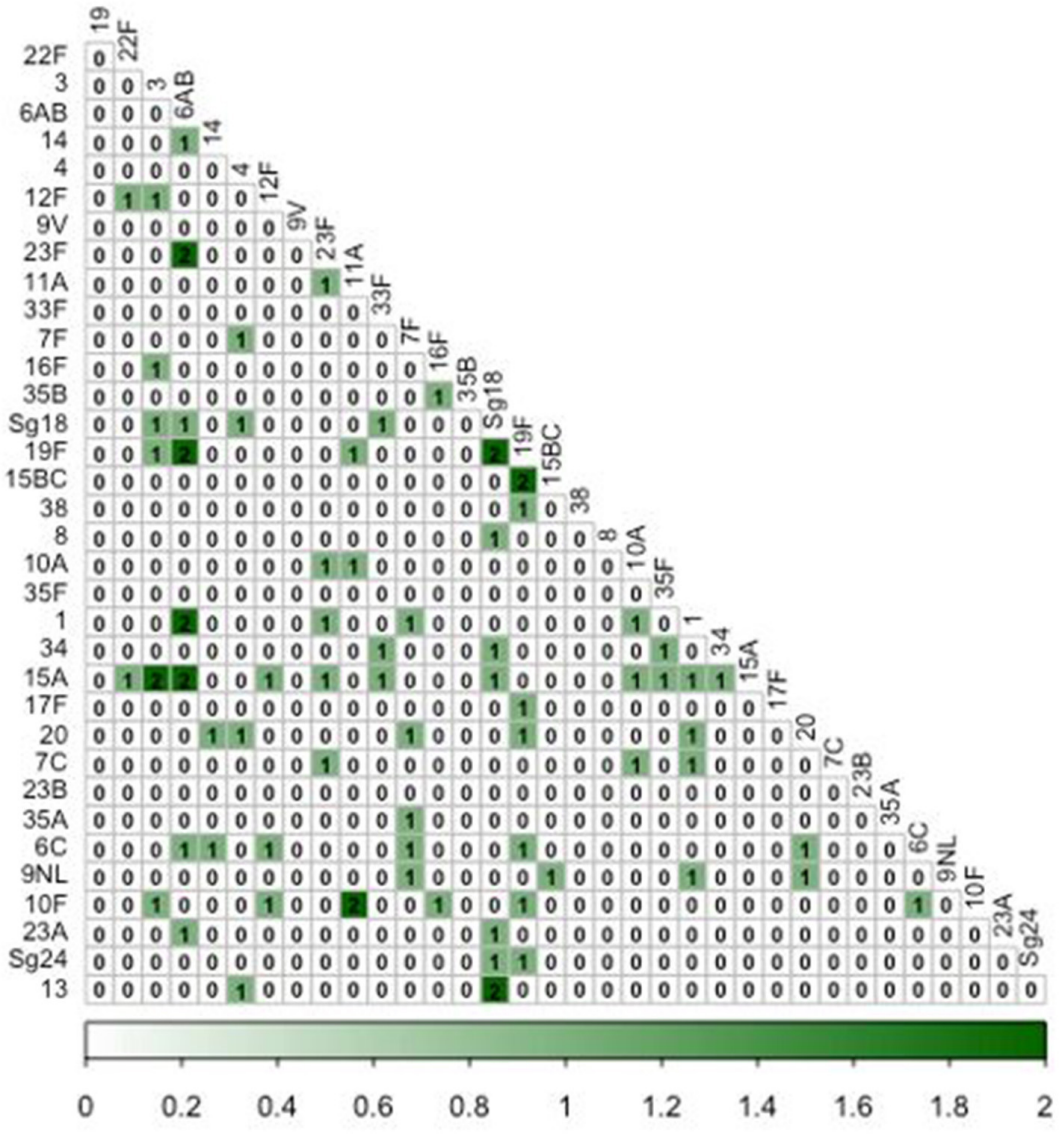
Co-occurrence matrix of pneumococcal serotypes among influenza-like illness cases. Numbers within cells represent the number of ILI episodes in which each pairwise combination of serotypes was detected.

More than one serotype was detected in 52 (19.3%) of the 269 pneumococcal-positive cases (Figure 3). Two serotypes were detected in the majority (41/52; 78.8%) of cases with serotype co-detection, while three serotypes were detected in nine cases (17.3%), and four in two cases (3.8%). Among pneumococcal-positive cases, prevalence of serotype co-detection was highest in cases under 5y (11/41; 26.8%), followed by 5–15y (24/124; 19.4%) and cases >15y (17/104; 16.3%), although this trend was not significant (${\chi^{2}}_{2}$ =2.07, *P* = 0.35).

### 3.5. Serotype recurrence among individuals with multiple ILI episodes

Throughout the surveillance period, 42 individuals experienced more than one pneumococcal-positive ILI episode (with a median 2, and range of 2 to 4 episodes across these individuals). However, repeat detections of the same serotype were observed in only nine (21.4%) of these individuals. The serotypes involved in these repeat detections were 6AB (*n* = 3 individuals), 11A (*n* = 2), 6C, 19A, 15A and 13. The median time interval between ILI episodes with a recurring serotype was 4.5 months (range: 1 to 36 months). It is unknown whether these events represented serotype persistence, or independent acquisitions at different time points.

### 3.6. Hospitalizations

Based on follow-up questionnaires (administered ~40 days after each ILI case investigation and sample collection), hospital admissions among ILI episodes were low (1.2%), and not significantly associated with pneumococcal positivity. Only three (1.1%) of the pneumococcal-positive ILI episodes were hospitalized; these cases were aged 4, 12, and 14 years, with serotypes 11A, 15A, and Sg18, respectively. The significance of *S. pneumoniae* detection in these cases is unclear; all three samples were also positive for at least one respiratory virus (influenza A/H3N2 in two cases, one of whom was also positive for seasonal coronavirus 229, while the third case was positive for respiratory syncytial virus). Their hospital stays were brief (~24 h), and all had fully recovered at the time of the follow-up visit. Official clinical diagnoses were not recalled by these participants or their relatives.

## 4. Discussion

Few studies have reported on pneumococcal detection and serotypes among ILI cases, particularly among cases identified through community-based surveillance as opposed to surveillance at healthcare facilities. In this study, we describe the prevalence and risk factors of nasopharyngeal pneumococcal detection among subjects with ILI in Lao PDR. In addition to higher prevalence in younger age groups, pneumococcal positivity was significantly associated with passive smoking and concurrent detection of *Hemophilus influenzae*. Detection of PCV13 serotypes was observed to decrease over the study period, although this trend was only significant in ILI cases under 5 y.

The inverse relationship between age and pneumococcal carriage has been widely reported elsewhere, including in studies among healthy children in Lao PDR (7) and other countries in Southeast Asia (17).

While the high prevalence of pneumococcal carriage in young children could implicate *S. pneumoniae* as an important etiological pathogen of ILI in this population, it is important to acknowledge that the clinical significance of pneumococcal detection among our study participants is unclear. *S. pneumoniae* bacteria commonly colonize the nasopharynx without leading to invasive disease, and ILI symptoms can be caused by a diverse range of viral and bacterial pathogens. We cannot provide evidence of a temporal association between ILI occurrence and pneumococcal acquisition/infection during the study period because only a single nasopharyngeal specimen was collected during the acute phase of ILI. Furthermore, we did not collect nasopharyngeal swabs in study participants without ILI. Thus, we refer to pneumococcal-positivity among ILI cases in our study as “carriage”, rather than pneumococcal infection or disease. While it is possible that some cases may have represented bacterial pneumonia initially presenting as ILI, our follow-up data on hospitalizations do not provide evidence support this. Hospitalizations among ILI cases were low, and not associated with *S. pneumoniae* detection. Moreover, all three pneumococcal-positive cases who were briefly admitted were also positive for at least one respiratory virus.

Consistently with other studies (18, 19), we identified passive smoking as a risk factor for pneumococcal detection. Exposure to cigarette smoke has been shown to impair nasal mucociliary clearance and increase pneumococcal adherence to respiratory epithelia (20–22), which may explain the associations observed in our study. Carriage of *Hemophilus influenzae* was also significantly associated with increased risk of pneumococcal positivity. A synergistic association between *S. pneumoniae* and *H. influenzae* colonization has been reported among Australian Aboriginal (23) and Italian children (24). The latter study also found that children vaccinated with PCV were even more likely to be colonized by *H. influenzae*. Commensal coexistence of these bacteria within a biofilm in the human nasopharynx has been suggested as a mechanism enabling their persistence in the host (25).

Similar to a study among hospitalized children with acute respiratory infection in Lao PDR between 2013 and 2019 (5), VT strains were found in 41% of pneumococcal-positive ILI cases, and accounted for 38% of all pneumococcal isolates detected. The significant decrease in VT detection between 2015/16 and 2018/19 among ILI cases aged < 5y was likely due to the impact of vaccination; all ILI cases in this age group that occurred in 2015/16 were born prior to October 2013 (i.e., before PCV13 introduction in Lao PDR), while the ILI cases aged < 5y in 2018/19 were born during or after this month, and therefore more likely to have been vaccinated. However, the relative contributions of direct vs. indirect effects of PCV13 on VT carriage in young children cannot be ascertained through our data. The cohort study was designed to investigate the etiology and epidemiology of ILI in the community, rather than PCV13 effectiveness in young children, and data on vaccination status of study participants were not available. Nonetheless, the temporal decrease in the proportion of VT isolates also seen among older age-groups (who would not have been vaccinated), while not statistically significant, may point to an emerging indirect (herd) effect due to decreasing circulation of PCV13 serotypes. Cross-sectional carriage surveys pre- and post-PCV have similarly reported evidence of the indirect effect of PCV13 in Lao PDR (5, 8). We also note that, despite the temporal decline in VT detection, we did not observe a significant change in overall pneumococcal prevalence during the study period. This may represent preliminary evidence of serotype replacement, although longer term studies are needed to further support this.

Similar to our results, serotypes 6A/B, and 19F have been found as the most prevalent PCV13 serotypes among 12–23-month-old Laotian children both before and after PCV13 introduction (8). The most common NVT serotypes in our study were 11A, 35B, 15A and 20. Serotypes 15A and 11A have been identified as among the top 10 serotypes causing invasive pneumococcal disease post-vaccination in France and Norway, respectively (16). Further investigation is warranted on the need for broader pneumococcal vaccines in Lao PDR, including the PPSV23, PCV15 or PCV20 which have been introduced, for example in older age groups, in other countries.

Two key limitations of our study are that, as discussed above, we are unable to draw strong conclusions regarding clinical significance of pneumococcal carriage in our study population, or the precise role of PCV13 introduction on changes in serotype distributions. Indeed, these reflect challenges associated with *S. pneumoniae* surveillance in ILI cases more generally. The use of serological assays (26, 27), alongside nasopharyngeal qPCR testing, could help elucidate the clinical significance of pneumococcal detection in future ILI studies. Nonetheless, our study illustrates how samples and data from community-based ILI surveillance can still be leveraged, for example to identify risk factors, circulating serotypes, and emerging trends in which may indicate serotype replacement. Another limitation is that serotype data could not be obtained for 21.1% of pneumococcal-positive swabs. *S. pneumoniae* screening was based on PCR detection of the *lyt A* gene, an important virulence marker which is thought to play a role in nasopharyngeal colonization (28). Realtime PCR targeting of *lyt A* is considered one of the gold standards among culture-independent *S. pneumoniae* assays, showing high diagnostic sensitivity and specificity (10, 11, 28). However, *lyt A* positivity has also been reported for some non-encapsulated pneumococci, as well as a small number of non-pneumococcal isolates (e.g., *Streptococcus pseudopneumoniae* carrying *lyt A* homologs) (10). Thus, while it seems likely that most of our “non-typeable” samples represent encapsulated *S. pneumoniae* serotypes (i.e., which were not included in the multiplex typing PCR), it is possible that some were due to non-encapsulated strains or even non-pneumococci, which are of lower clinical and vaccine importance. We also acknowledge the possibility that variation in detection limits of the serotyping assay for different strains could have had some influence on the observed serotype and NT frequencies (12).

## Conclusion

In conclusion, we found pneumococcal carriage among ILI cases in Vientiane to be associated with younger age, passive smoking, and concurrent *H. influenzae* colonization. Our findings contribute also to the growing body of evidence from low- and middle-income countries indicating the effect of PCV13 on serotype distributions. Analysis of *S. pneumoniae* carriage data among ILI cases detected through community-based surveillance, such as the LaCORIS study, can complement data from surveillance at health facilities, to provide a more complete picture of pneumococcal burden and circulating serotypes across all age-groups, including associations with other respiratory pathogens. Such evidence can help inform future studies aiming to evaluate the impact of vaccination on community transmission, and identify which serotypes may be important to consider in future vaccines.

## Data availability statement

The datasets presented in this article are not readily available because it is confidential information that will require the authorization of several study partners including the Funder, MoH, and Ethics Committee. Requests to access the datasets and R scripts should be directed to the corresponding author VSP.

## Ethics statement

The studies involving human participants were reviewed and approved by the Lao National Ethics Committee for Health Research in Lao PDR (060/NECHR; 1 January 2017). Written informed consent to participate in this study was provided by the participants' legal guardian/next of kin.

## Author contributions

VSP designed the study. IK, PP, and BF coordinated and supervised data collection. JR analyzed the data. MS-E, JR, and VSP drafted the manuscript. All authors reviewed and approved the final manuscript.
