# Supplementary material for: Epidemiology and serotype distribution of Streptococcus pneumoniae carriage among influenza-like illness cases in metropolitan Vientiane, Lao PDR: a community-based cohort study
Source: Front Public Health. 2023 Apr 20;11:1124016. doi: 10.3389/fpubh.2023.1124016 (PMC10157285; doi:10.3389/fpubh.2023.1124016)
Supplement: Supplementary file 1 [file Table_1.DOCX]

Supplementary Material

**Epidemiology and serotype distribution of *Streptococcus pneumoniae* carriage among influenza-like illness cases in metropolitan Vientiane, Lao PDR: a community-based cohort study**

**Valentina Sanchez Picot^1^*,** **Inthalaphone** **Keovichith^2^, Phimpha Paboriboune^2^, Bruno Flaissier^3^, Mitra Saadatian-Elahi^4, 5^, James W. Rudge^6,7^**

^1^ Fondation Mérieux, 17 rue Bourgelat, 69002 Lyon-France

^2^ Center of Infectiology Christophe Mérieux of Laos, Samsenthai Road, Kaoyot Village, Sisathanak District, Vientiane Capital, Lao P.D.R

^3^ Fondation Mérieux, Laos

^4^ Service Hygiène, Epidémiologie et Prévention. Centre Hospitalier Hôpital Eduard Herriot, Hospices Civils de Lyon, 69437 Lyon Cedex, France

^5^ CIRI, Centre International de Recherche en Infectiologie, (Team Public Health, Epidemiology and Evolutionary Ecology of Infectious Diseases (PHE3ID)), Univ Lyon, Inserm, U1111, Université Claude Bernard Lyon 1, CNRS, UMR5308, ENS de Lyon, F-69007, Lyon, France.

^6^ Communicable Diseases Policy Research Group, Department of Global Health and Development, London School of Hygiene & Tropical Medicine, UK.

^7^Faculty of Public Health, Mahidol University, Bangkok, Thailand.

*** Correspondence :**Dr Valentina Sanchez Picot
[valentina.picot@fondation-merieux.org](mailto:valentina.picot@fondation-merieux.org)

# Supplementary Tables

**Supplementary Table 1.** Bivariable and multivariable analyses for associations with nasopharyngeal pneumococcal detection among influenza-like illness cases (n=, after removal of data on repeat ILI episodes within the same individuals. (This supplementary analysis was conducted to check that random effects at individual level were not substantially influencing the associations shown in Table 1 of the main manuscript.)

|  |  |  | Bivariable analysis | | Multivariable analysis | |
| --- | --- | --- | --- | --- | --- | --- |
| Variable | Level | Pneumococcal carriage n/N (%) | Crude OR^a^  (95% CI) | *P* | Adjusted OR^a^  (95% CI) | *P* |
| Age group (y) | 0-4 | 30/54 (55.6) | ref |  | ref |  |
|  | 5-15 | 88/217 (40.6) | 0.43 (0.20,0.93) | 0.032 | 0.51 (0.23,1.14) | 0.010 |
|  | 16+ | 64/845 (7.6) | 0.04 (0.02,0.10) | <0.001 | 0.05 (0.02,0.13) | <0.001 |
| Sex | Female | 101/637 (15.9) | ref |  | ref |  |
|  | Male | 81/479 (16.9) | 1.09 (0.76,1.55) | 0.642 | 0.94 (0.63,1.41) | 0.762 |
| Year | 2015/16 | 62/384 (16.1) | ref |  |  |  |
|  | 2017/18 | 66/376 (17.6) | 1.06 (0.69,1.64) | 0.776 |  |  |
|  | 2018/19 | 54/356 (15.2) | 0.91 (0.58,1.42) | 0.668 |  |  |
| Season | Dry | 92/549 (16.8) | ref |  |  |  |
|  | Wet | 90/567 (15.9) | 0.91 (0.63,1.30) | 0.592 |  |  |
| Residence area | Urban | 38/246 (15.4) | ref |  |  |  |
|  | Peri-urban | 62/402 (15.4) | 0.94 (0.55,1.61) | 0.832 |  |  |
|  | Suburban | 82/468 (17.5) | 1.19 (0.71,1.97) | 0.508 |  |  |
| SES category | Lowest | 57/366 (15.6) | ref |  |  |  |
|  | Medium | 48/380 (12.6) | 0.79 (0.48,1.30) | 0.355 |  |  |
|  | Highest | 77/370 (20.8) | 1.58 (0.99,2.51) | 0.056 |  |  |
| HH crowding  (Persons per sleeping room | <2.5 | 107/779 (13.7) | ref |  | ref |  |
|  | ≥2.5 | 75/337 (22.3) | 1.96 (1.29,2.98) | 0.001 | 1.49 (0.95,2.35) | 0.086 |
| No. of children <5y in HH | 0-1 | 164/1054 (15.6) | ref |  | ref |  |
|  | 2 or more | 18/62 (29.0) | 2.78 (1.30,5.98) | 0.008 | 1.14 (0.48,2.71) | 0.756 |
| No. of children  5-15y in HH | 0-1 | 115/826 (13.9) | ref |  | ref |  |
|  | 2 or more | 67/290 (23.1) | 1.96 (1.29,2.99) | 0.001 | 1.08 (0.67,1.75) | 0.747 |
| Exposure to HH cigarette smoke | No | 83/608 (13.7) | ref |  | ref |  |
|  | Yes | 99/508 (19.5) | 1.57 (1.06,2.30) | 0.022 | 1.62 (1.05,2.49) | 0.028 |
| Chronic condition | No | 169/965 (17.5) | ref |  | ref |  |
|  | Yes | 13/151 (8.6) | 0.43 (0.22,0.81) | 0.009 | 1.11 (0.55,2.24) | 0.765 |
| *S. aureus* | No | 154/974 (15.8) | ref |  |  |  |
|  | Yes | 28/142 (19.7) | 1.40 (0.83,2.34) | 0.204 |  |  |
| *C. pneumoniae* | No | 179/1108 (16.2) | ref |  | ref |  |
|  | Yes | 3/8 (37.5) | 3.86 (0.68,21.86) | 0.127 | 1.50 (0.25,9.03) | 0.660 |
| *M. pneumoniae* | No | 176/1095 (16.1) | ref |  |  |  |
|  | Yes | 6/21 (28.6) | 2.35 (0.75,7.32) | 0.140 |  |  |
| *H. influenzae* | No | 173/1102 (15.7) | ref |  | ref |  |
|  | Yes | 9/14 (64.3) | 15.95 (4.05,62.87) | <0.001 | 4.79 (1.15,20.02) | 0.032 |
| Respiratory virus (any) | No | 47/337 (13.9) | ref |  |  |  |
|  | Yes | 135/779 (17.3) | 1.29 (0.86,1.93) | 0.210 |  |  |
| Influenza A | No | 153/938 (16.3) | ref |  |  |  |
|  | Yes | 29/178 (16.3) | 0.97 (0.60,1.58) | 0.905 |  |  |
| Influenza B | No | 163/1015 (16.1) | ref |  |  |  |
|  | Yes | 19/101 (18.8) | 1.23 (0.67,2.24) | 0.507 |  |  |
| Coronavirus (seasonal) | No | 155/955 (16.2) | ref |  |  |  |
|  | Yes | 27/161 (16.8) | 1.05 (0.64,1.75) | 0.834 |  |  |
| Rhinovirus | No | 146/918 (15.9) | ref |  |  |  |
|  | Yes | 36/198 (18.2) | 1.16 (0.73,1.83) | 0.526 |  |  |
| Parainfluenza | No | 167/1036 (16.1) | ref |  |  |  |
|  | Yes | 15/80 (18.8) | 1.33 (0.68,2.61) | 0.402 |  |  |

^a^OR=odds ratio. Crude ORs are adjusted for random effects at household level. Adjusted ORs are derived from a generalized linear mixed model which included age, sex, and all covariates which showed significance of P<0.05 in bivariable analyses (i.e., adjusting for all the other covariates in the Table for which adjusted ORs are shown, in additional to random effects at household level).

**Supplementary Table 2.** Simpson’s Diversity Indices for pneumococcal serotypes detected among *S. pneumoniae-*positive ILI cases by age group and surveillance year.

| Variable | Level | Simpson’s Diversity Index (95% CI) |
| --- | --- | --- |
| Age group (y) | 0-4 | 0.949 (0.920-0.978) |
|  | 5-15 | 0.958 (0.947-0.968) |
|  | 16+ | 0.946 (0.929-0.964) |
| Year | 2015/16 | 0.945 (0.924-0.966) |
|  | 2017/18 | 0.949 (0.934-0.964) |
|  | 2018/19 | 0.959 (0.941-0.978) |
